# Optimal site selection strategies for urban parks green spaces under the joint perspective of spatial equity and social equity

**DOI:** 10.3389/fpubh.2024.1310340

**Published:** 2024-04-04

**Authors:** Youqiang Zhao, Peng Gong

**Affiliations:** College of Gardening and Arts, Jiangxi Agricultural University, Nanchang, China

**Keywords:** urban park green spaces, park quality, Gaussian two-step floating catchment area, accessibility, spatial equity, social equity

## Abstract

Urban park green spaces (UPGS) are a crucial element of social public resources closely related to the health and well-being of urban residents, and issues of equity have always been a focal point of concern. This study takes the downtown area of Nanchang as an example and uses more accurate point of interest (POI) and area of interest (AOI) data as analysis sources. The improved Gaussian two-step floating catchment area (G2SFCA) and spatial autocorrelation models are then used to assess the spatial and social equity in the study area, and the results of the two assessments were coupled to determine the optimization objective using the community as the smallest unit. Finally, the assessment results are combined with the k-means algorithm and particle swarm algorithm (PSO) to propose practical optimization strategies with the objectives of minimum walking distance and maximum fairness. The results indicate (1) There are significant differences in UPGS accessibility among residents with different walking distances, with the more densely populated Old Town and Honggu Tan areas having lower average accessibility and being the main areas of hidden blindness, while the fringe areas in the northern and south-western parts of the city are the main areas of visible blindness. (2) Overall, the UPGS accessibility in Nanchang City exhibits a spatial pattern of decreasing from the east, south, and west to the center. Nanchang City is in transition towards improving spatial and social equity while achieving basic regional equity. (3) There is a spatial positive correlation between socioeconomic level and UPGS accessibility, reflecting certain social inequity. (4) Based on the above research results, the UPGS layout optimization scheme was proposed, 29 new UPGS locations and regions were identified, and the overall accessibility was improved by 2.76. The research methodology and framework can be used as a tool to identify the underserved areas of UPGS and optimize the spatial and social equity of UPGS, which is in line with the current trend of urban development in the world and provides a scientific basis for urban infrastructure planning and spatial resource allocation.

## 1 Introduction

Urban densification has emerged as a prevailing global development trend (1), wherein the concentration of buildings and populations exacerbates the conflict between the provision of public resources and population demands (2). Particularly in China, ensuring an equitable and just allocation of social public resources has become a pivotal focus in formulating urban development plans by the Chinese government (3). Urban park green spaces (UPGS), as the fundamental building block of social public resources, carry many benefits for urban ecology, economy, and society (4–6). They provide urban residents with places for daily activities are important in enhancing the health and well-being of urban residents, and are regarded as a key factor in ensuring their physical health and well-being (7). However, in high-density urban environments, the mismatch between social supply and demand constrains the fairness of residents’ enjoyment of UPGS resources and undermines their right to enjoy social public resources equally (8). Therefore, it is of great significance to study the supply–demand relationship of UPGS and optimize the spatial layout of UPGS to improve the well-being of residents, promote the fairness of supply–demand, and the sustainable development of the city.

The study of UPGS equity has its origins in the “environmental equity” movement in the United States (9). Up to now in development, research on UPGS equity measures has undergone three stages: territorial equality, spatial fairness, and social fairness (10). Among these stages, territorial equality emphasizes the equitable distribution of UPGS quantity and area in the macro-geographical space (11). Spatial equity introduces the important indicator of accessibility, which reflects the interrelationship between the supply of UPGS and the demand of the population, intending to seek a balance between the two (12). Social fairness primarily addresses disparities in UPGS service levels among different types of residents, shifting focus from objects to individuals (13). Overall, UPGS equity research has evolved from the initial geographical parity to the use of accessibility modeling to explore spatial equity, to social equity that simultaneously considers spatial layout, group differences, and human needs (14). At this stage, research has focused on the relationship between accessibility equity and resident attributes, such as the fact that racial minority communities have less access to UPGS resources and recreational programs than white communities in some racially discriminatory US cities (15) and that the poor have less access to UPGS than the rich in cities with uneven regional economic development (16, 17). In addition, statistical analyses of major cities in Germany have shown differences in access to UPGS between groups of different genders and different levels of education (18); some studies in China have also disclosed that individual physical factors can lead to greater resistance to accessing high-quality UPGS for disadvantaged groups, such as children, the older adult, and pregnant women (19, 20). Thus, it can be seen that there are large differences in accessibility equity and resident attributes under different cities, and research on UPGS equity under different cities is also necessary.

Accessibility serves as a core measure for spatial equity and social fairness, which was first introduced in 1948 as a measure of “human participation potential” (21) and has progressively evolved into a critical reference factor for UPGS planning (22). Traditional approaches to measuring UPGS accessibility include methods such as the minimum distance approach (23), buffer analysis (24), gravity modeling (25), network analysis (26), and the two-step floating catchment area (2SFCA) (27). Among them, both the minimum distance method and buffer analysis method do not consider the actual road network. The former uses Euclidean distance as a criterion to calculate the straight-line distance from residents to supply points, while the latter employs a predetermined search radius to identify the number and area of public facilities within that radius or calculate the number of settlements within a certain service radius of a public facility (28). On the other hand, although the network analysis method considers the actual road network by calculating service range at a predetermined time or distance based on supply points, it fails to account for supply–demand relationships. In contrast, the gravity model method measures spatial accessibility by summing up probabilities associated with multiple facility choices at each demand location, taking into consideration attraction, supply and demand impacts as well as spatial friction. However, this approach requires complex data, and determining resistance coefficients is challenging (29). Based on two searches centered around demand and supply points, respectively, using road networks, 2SFCA builds upon the gravity model method to determine convenience between supply and demand. It also integrates urban public facility scale, demand scale, and distance relationship between supply and demand (30). Nevertheless, this method does not consider distance attenuation but defaults to assigning an equal probability of choice for residents within the same search range (28). To solve these problems, scholars have introduced various forms of extensions into the 2SFCA model, and the main directions of the extensions include the introduction of different search radii (14), the introduction of differential traveling modes (31), and the introduction of geographic impedance decay functions (32). Enhanced 2SFCA based on 2SFCA (33), Variable 2SFCA (34) Gaussian 2SFCA (G2SFCA) (35), and so on appeared. Among them, G2SFCA, compared to the other two improved models, introduces Gaussian equations based on 2SFCA, which not only takes into account the spatial barrier between supply and demand points but also captures the phenomenon that people’s willingness to travel gradually decreases with the increase of distance. Therefore, its accessibility results are closer to the real situation (36, 37). Thus, after comparing the existing accessibility measurement models, we chose G2SFCA, which considers the supply–demand equilibrium and attenuation distance, as our measurement model to be improved. However, previous studies on improving UPGS reachability models and measurements have some limitations. One of the main limitations is that they mainly focus on factors such as traveling mode, distance, and environmental resistance while neglecting the impact of UPGS quality attributes on residents’ probability of choice and visitor capacity. Closer and larger UPGS facilities tend to attract more visitors and provide a better experience for residents, and some UPGS infrastructures can also have limitations on visitor numbers and visitor experience (36, 38). Therefore, incorporating multidimensional UPGS quality attributes into the accessibility measurement model can help to comprehensively capture real-life accessibility resistance, as well as comprehensively reveal the rationality and fairness of UPGS layout (39). In addition, the degree of influence of UPGS quality attributes on the level of accessibility varies widely. With reference to the relevant literature, we eliminated subjective attributes such as environmental quality and landscape beauty, and finally refined the UPGS quality attributes with more objective and influential ecological service value (ESV) and recreational facility capacities (FCs), thus proposing a more accurate G2SFCA model (38).

Currently, most studies on the equity of urban parks and green spaces focus on social equity (14), assessing the reasonableness of the layout of UPGS by examining the degree of accessibility for specific groups of residents. However, no matter which attribute of residents is chosen as the research object, there is a specificity of the research results, which limits the generality and applicability of the research conclusions to be applied in the construction of urban public space systems. In contrast, spatial equity research tends to take large-scale non-specific groups as research objects, making it difficult to accurately grasp the needs of socially disadvantaged groups. Combining these two perspectives can solve the spatial equity problems of ordinary residents with minimal computational costs while taking into account the social equity problems of special groups, providing a new direction for optimizing the equity problems in the construction of UPGS. As an effective tool for socio-economic differentiation, house prices cover all urban populations, creating opportunities for the combination of spatial and social equity. In addition, existing social equity studies have only analyzed differences in access to UPGS resources from the perspective of different demographic attributes, ignoring the social inequity situation caused by differences in economic structure. Exploring the correlation analysis between community house prices and UPGS accessibility can effectively fill this gap (36). Particularly in China, house prices promote socioeconomic redistribution (40), leading to a homogeneous agglomeration pattern of residents with similar economic conditions in a given urban area (41–43). On this basis, this study attempts to quantitatively assess the equity of access to urban public infrastructure services for different socio-economic groups through spatial autocorrelation analyses of house prices and accessibility in a community in Nanchang, China, and to filter out the optimization objectives under social equity. Then, the UPGS accessibility levels of all residents were stratified and the optimization objectives under spatial equity were screened. Finally, using the K-means algorithm and Particle Swarm Algorithm (PSO) with minimum walking distance and maximum fairness as the optimization principles, the two objectives under this spatial and social fairness are used as optimization objects, and combined with the green space information in the local urban land-use plan, the location and area of parks to be built are clarified, which provide a reference for the fair distribution of high-density urban public facilities.

**Figure 1:**
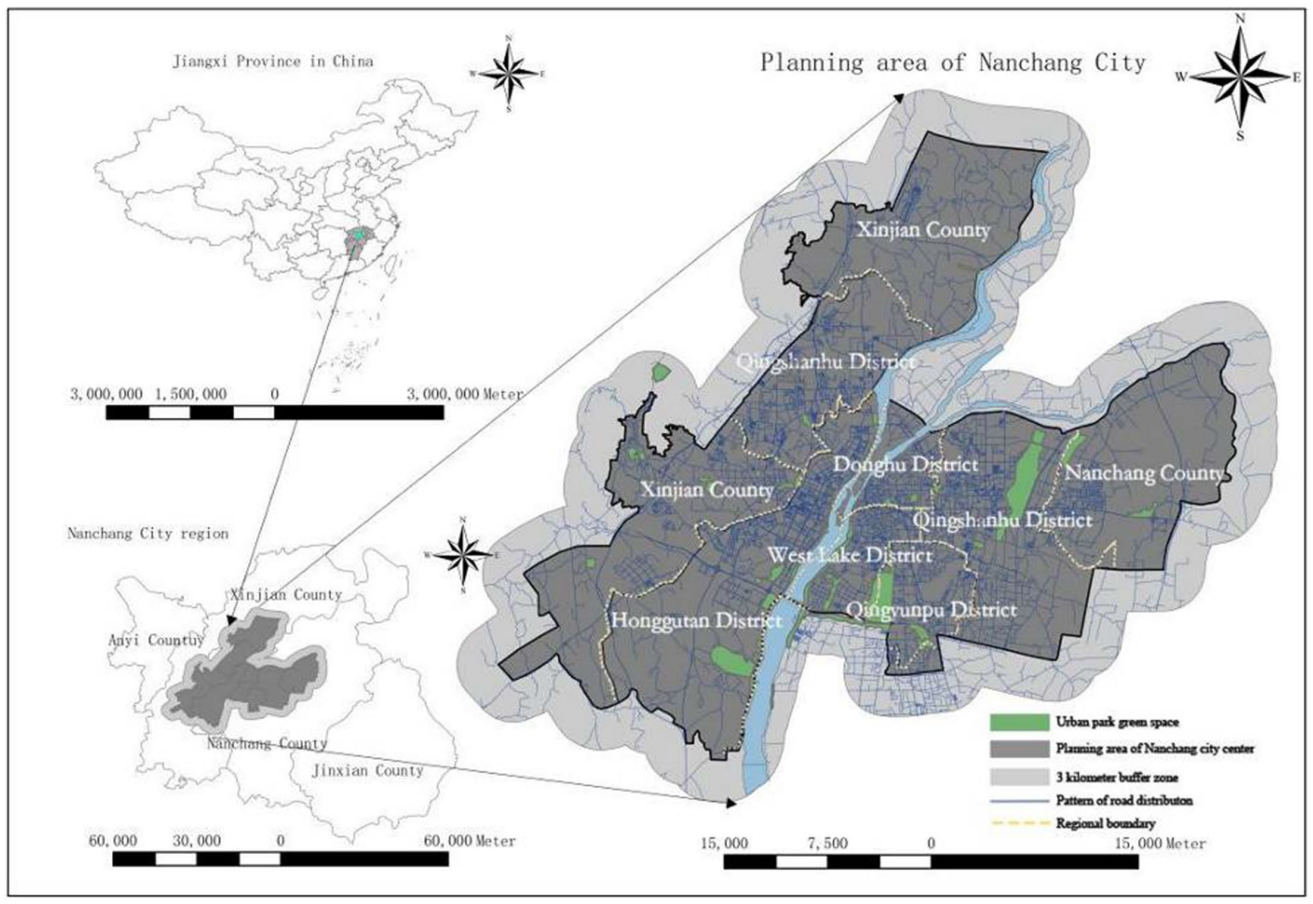
The location of Nanchang City in China, the research area in Nanchang City, and the spatial distribution of UPGS.

Taken together, the new UPGS optimization scheme proposed in this paper consists of three main parts. (1) Introducing the more objective UPGS quality attribute to improve the G2SFCA model, aiming to maximize the replication of real-life UPGS accessibility resistance. (2) Incorporating a social fairness perspective to determine the number of settlements to be optimized and their distribution through the coupling of spatial and social fairness. (3) Through the combination of the K-means algorithm and PSO as well as the reference of land use planning layout in the study area, new UPGS locations, and areas are created for the settlements to be optimized to guide urban planning (See Figure 1).

## 2 Study area and research data

### 2.1 Study area

Earlier research cases on UPGS equity within China have focused on developed coastal cities in the Yangtze River Delta and Pearl River Delta, such as Shanghai (44, 45) and Shenzhen (46, 47). With the increasing imbalance between the supply and demand of public service resources within each city and the advancement of regional economic integration, the research has been extended to inland cities such as Changsha (41) and Wuhan (19). The expansion of the study cases from developed to sub-developed regions is in line with the global trend in public resources research. This group of landlocked cities, which are economically more underdeveloped and suffer from a significant imbalance between the supply of and demand for public service resources, needs to be given greater attention, as they account for a greater number of cities globally (48).

For our case study, we chose one of China’s most representative inland cities, Nanchang, the capital of Jiangxi Province. This UPGS study is directly related to the development of Nanchang’s livelihoods, as Nanchang is trying to address issues such as uneven distribution of public resources and residential segregation (49), while actively developing a UPGS service that is highly relevant to the city’s inhabitants, namely the ‘Walking Living Circle’. According to statistics from the Nanchang City Planning Bureau, by the end of 2022, the *per capita* area of park green space in Nanchang will be 13.05 square meters, which is significantly lower than the national average of 14.87 square meters *per capita*. The large population base coupled with the influx of a large number of foreigners has exacerbated the conflict between supply and demand for UPGS in Nanchang (48). Geographically, it spans from east longitude 115°27′ to 116°35′ and from north latitude 28°10′ to 29°11. This study takes the planning blueprint presented by the Nanchang Urban Planning Bureau as the research area, with a total area of 1,005 km^2^, including Donghu District, Xihu District, Honggutan District, Qingyunpu District, Qingshanhu District, Xinjian District, and parts of Nanchang County. Among these, Donghu District, Xihu District, and parts of Qingshanhu District constitute the old city center of Nanchang. Additionally, considering that residents near the boundaries of the study area may use UPGS beyond the internal regions, UPGS within a 3 km buffer zone around the research area is also included for analysis purposes.

### 2.2 Data sources and processing

#### 2.2.1 Park data

**Figure 2:**
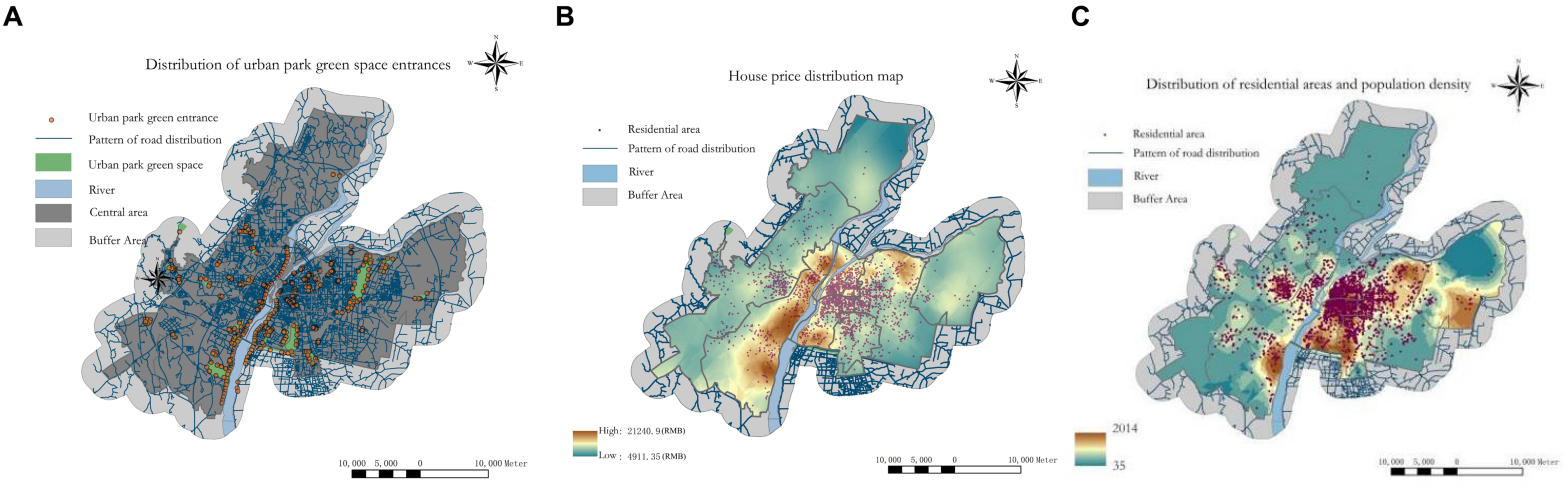
The UPGS inlet distribution **(A)**, the house price distribution map **(B)**, and the distribution of residential areas and population density **(C)**.

First, Python programming was employed to scrape the Point of Interest (POI) directory for UPGS in Nanchang City in 2023. Subsequently, the Baidu Maps open API was utilized to retrieve the Area of Interest (AOI) data for UPGS. After eliminating charged or abandoned parks and those with overlapping areas, 91 UPGS were acquired. Following the “Nanchang Urban Green Space System Planning (Revised) (2015–2020)” and “England Natural Green Space Accessibility Guidelines - Natural Green Space Accessibility Standards,” the UPGS within the research area were categorized into four groups based on area parameters: comprehensive parks [≥25 hm^2^, (21)], citywide parks [5–25 hm^2^, (44)], regional parks [2–5 hm^2^, (14)], and community parks [<2 hm^2^, (12)]. Referring to the existing literature on park service radii (50, 51), walking service radii for community, regional, citywide, and comprehensive UPGS were set as follows: 500 m, 1,000 m, 2,000 m, and 3,000 m, respectively. Furthermore, considering that park entrances and exits are more scientifically relevant supply points than their geographical centers (52, 53), 394 entries and exits for UPGS were identified through Google Maps recognition and on-site surveys (Figure 2A). Among these parks, due to the elongated nature of greenway-type UPGS and the absence of barriers such as walls or hedges in most areas, intersections between greenways and main roads were transformed into supply points (54).

#### 2.2.2 Road data

Download the latest road data for Nanchang City by accessing the OpenStreetMap website,^1^ and after topological checks, road network matching, data correction, and elimination import it into ArcMap 10.6 for analysis.

#### 2.2.3 Housing and population data

The Housing POI data were collected from Anjuke, one of China’s largest platforms for second-hand housing transactions.^2^ This dataset includes essential information such as the names of housing communities, latitude and longitude coordinates, construction year, community household numbers, and housing prices (price per square metre in RMB). After thorough filtering and cleaning processes, 3,024 residential points were obtained. Previous studies have demonstrated that utilizing housing unit numbers and prices as proxies for regional population distribution and residents’ economic conditions can significantly enhance the scientific validity and reliability of UPGS fairness measurements (55, 56). Therefore, we employed community housing prices to indicate residents’ financial status (Figure 2B). At the same time, the total population was estimated by multiplying the total number of households with administrative unit population data (Figure 2C). The population data for administrative units were sourced from the sixth national population census at the block level in Nanchang City (Nanchang Statistical Bureau).

## 3 Research methodology

### 3.1 Improvements to GS2SFCA and accessibility measurement

The G2SFCA method, which incorporates distance decay and supply factors, is selected for computation in this study. By integrating ESV and FCs derived from park quality into the traditional formula, a more precise G2SFCA accessibility measurement model is developed. The specific calculation involves three sequential steps.

The first step involves calculating the contribution ratio of the comprehensive supply capacity of UPGS, which is based on the area (S), ESV, and FCs. The study shows that the fragmentation of the green landscape under urban space is an important aspect of measuring the ecological service value of the UPGS, i.e., the more fragmented the green space is, the worse the ecological service value and the social service function are (57). The landscape pattern index, as a method to quantitatively study the pattern characteristics, can effectively respond to the degree of green space fragmentation within the UPGS. Referring to related studies, three major hierarchical indexes describing patches under landscape pattern: patch spatial layout, patch shape, and patch area and density were introduced for assessing the degree of fragmentation of Greenland patches within the UPGS (58), in which the extraction of the degree of the landscape of Greenland patches was based on based on China’s first set of 1 m-resolution nationwide land cover maps (SinoLC-1) (59). Specifically, mesh size (MESH), split index (SPLIT), aggregation index (AI) as measures of patch spatial arrangement; weighted patch area size (AREA_AM), average shape index (SHAPE_MN), division index (DIVISION) as measures of patch shape; and patch density (PD), landscape patch index (LPI), and landscape shape index (LSI) as measures of patch area and density. Among them, AI, LPI, and MESH are positive indices to evaluate the quality of green space patches; PD, LSI, AREA_AM, SHAPE_MN, DIVISON and SPLIT are negative indices to evaluate green space patches there. After extracting each landscape pattern index, each index was standardized with positive and negative values, and then integrated using principal component analysis in SPSS, and finally obtained the ESV value of each UPGS and the average value of different types of UPGS: community-type (77.53) > citywide type (42.74) > comprehensive type (7.51) > regional type (−8.26) (Table 1). Furthermore, it has been well-documented that the carrying capacity of FCs, as a crucial quality attribute of UPGS, directly influences residents’ willingness to visit (36, 38) as the central accommodation for FCs within UPGS, the area of hard surfaces is positively correlated with the service capacity of open spaces in most cases (60). Therefore, in this study, the scope of hard grounds in the UPGS within the study area can be extracted as an indicator for estimating the carrying capacity of FCs using the raster calculator in ArcMap 10.6.

**Table 1:** Weights of GS2FCA-related indicators after improvement.

Related Indicator	Description	Weights
S	Area size of UPGS	0.7236
FCs	Hard site area of UPGS	0.1931
ESV	E1(comprehensive type) = 7.51, E2(city-wide type) = 42.74, E3(regional type) = −8.26, E4(community type) = 77.53	0.0833

Eventually, a judgment matrix based on the De Feur method was created through AHP (hierarchical analysis) (61) to determine the weights of park S, ESV, and FCs (Table 1). Additionally, the consistency ratio (CR) value obtained from this judgment matrix consistency test was found to be 0.08, below 0.1, and thus passed the one-time test (62).

The second step involves computing the service capacity *R_j_* for each UPGS. A corresponding spatial influence domain is established using each entrance and exit of a UPGS as a supply point *j*, with *j* as the center and selecting *d*_0_ as the search radius. Weights are assigned using the Gaussian equation for all resident demand points *k* within this domain. Subsequently, the supply–demand ratio *R_j_* is calculated by dividing the comprehensive supply capacity of UPGS in terms of *S*, ESV, and FCs by the population of the weighted demand points (Eq. 1).

(1) $$R_{j}=\frac{S_{j}w_{1}+{ESV}_{j}w_{2}+{FCs}_{j}w_{3}}{\underset{k\in \;\left\{d_{kj}\leq d_{0}\right\}}{\sum }\left[G\left(d_{kj},d_{0}\;\right)\;P_{k}\right]}$$

Where *R_j_* is the supply–demand ratio, *S_j_*, ESV*_j_*, and FCs*_j_* are the total area, ecological service value, and recreational facilities of the *j*th UPGS, respectively; *W*_1_, *W*_2_, and *W*_3_ are the weights belonging to the above variables, respectively; *d*_0_ denotes the search threshold; *d_kj_* is the actual walking distance from the demand point k to the supply point *j*; *P_k_* is the total population in the role of the domain of the residents at the demand point *k*; and *G*(*d_kj_,d*_0_) is the distance decay function, and the calculation formula is shown in Eq. 2:

(2) $$G\left(d_{kj},d_{0}\right)=\{\begin{array}{l} \frac{e^{-\frac{1}{2}\times {\left(\frac{d_{kj}}{d_{0}}\right)}^{2}}-e^{-\frac{1}{2}}}{1-e^{-\frac{1}{2}}},d_{kj}\leq d_{0}\\ 0,d_{kj}\geq d_{0} \end{array}$$

In the third step, the accessibility index for each demand point is computed. Taking demand point *i* as the search center, the supply–demand ratios *R_j_* of all UPGSs in the *d*_0_ spatial domain are weighted and summed by the Gaussian function, and finally, the accessibility *A_i_* of demand point *i* is obtained (Eq. 3).

(3) $$A_{i}=\underset{j\in \left\{d_{ij}\leq d_{0}\right\}}{\sum }\left[g\left(d_{kj},d_{0}\right)R_{j}\right]$$

### 3.2 Spatial autocorrelation

To identify the mismatch between the accessibility of UPGS and residents’ socioeconomic levels, we utilized the GeoDa software. We employed the bivariate local Moran’s index as a measure of spatial autocorrelation (63), as shown in Eq. 4:

(4) $$I_{xy}^{i}=z_{x}^{i}\overset{n}{\underset{j=1}{\sum }}w_{ij}z_{y}^{j}$$

In the equation, represents the standardized value of the independent variable *x* (UPGS accessibility) for region *i*, $Z_{y}^{j}$ represents the standardized value of the dependent variable *y* (community house prices) for region *j*, and *w_ij_* is the spatial weight matrix between regions *i* and *j*. The measurement results of the bivariate local Moran’s index indicate five types of spatial local associations between UPGS accessibility and residents’ socioeconomic status: H-H (high accessibility, high socioeconomic level), H-L (high accessibility, low socioeconomic level), Not significant (both variables are not essential), L-H (low accessibility, high socioeconomic status), and L-L (low accessibility, low socioeconomic level).

### 3.3 Identification of supply blind zones

Supply blind zones refer to areas where the supply capacity of UPGS cannot adequately meet the resident’s needs, and they are categorized into explicit blind zones and implicit blind zones (64). Detailed blind zones indicate regions where residents’ distribution points cannot reach any UPGS within a 3,000 m walking distance. Implicit blind zones refer to areas where the service range of UPGS covers residents’ moments. Still, supply imbalances occur due to high population density or insufficient UPGS capacity, resulting in residents receiving a lower level of UPGS services.

### 3.4 Optimisation of supply blind zones

The optimization of the supply blind zones is based on the Nanchang Land Use Master Plan and uses a combination of the k-means algorithm and the PSO algorithm. The former determines the minimum number of UPGS required for these blind zones, and the latter determines the optimal location of UPGS clusters. Among various clustering algorithms, the k-means algorithm stands out for its interpretability, simplicity, and efficiency when dealing with large-scale datasets (65). We use this algorithm to cluster the main factors affecting spatial and social equity, including spatial accessibility based on a neighborhood scale and the degree of spatial matching of socio-economic levels, and the number of clusters is determined based on the maximum Euclidean distance of the paired samples in the two-dimensional space consisting of both. The PSO algorithm is a simulation of a simple social system such as the foraging behavior of birds and is achieved through an iterative process of Global optimisation. The algorithm has the advantages of simple implementation, high accuracy, and fast convergence (66). The PSO algorithm in this study first needs to generate a certain number of particles based on the clustering results of the K-means algorithm, with each particle representing a candidate UPGS siting scheme, and then calculate the fitness of each particle by using the sum of the products of the nearest neighbors distances between all particles as the objective function. Then, during multiple iterations, we update the velocity and position of each particle using the information of global optimum and individual optimum until a predetermined iteration limit or stopping condition is reached. The spatial location information of a certain amount of particle numbers is finally obtained as the UPGS pre-siting scheme. However, it is difficult to take into account the land use information of the city in the PSO algorithm, so the final optimization scheme is based on the principles of minimum walking distance and maximum fairness, which is refined and evaluated by the manual visual method knot and the results of the PSO algorithm with the land use information of Nanchang City.

## 4 Results analysis

### 4.1 Analysis of accessibility differences at different walking distances

**Figure 3:**
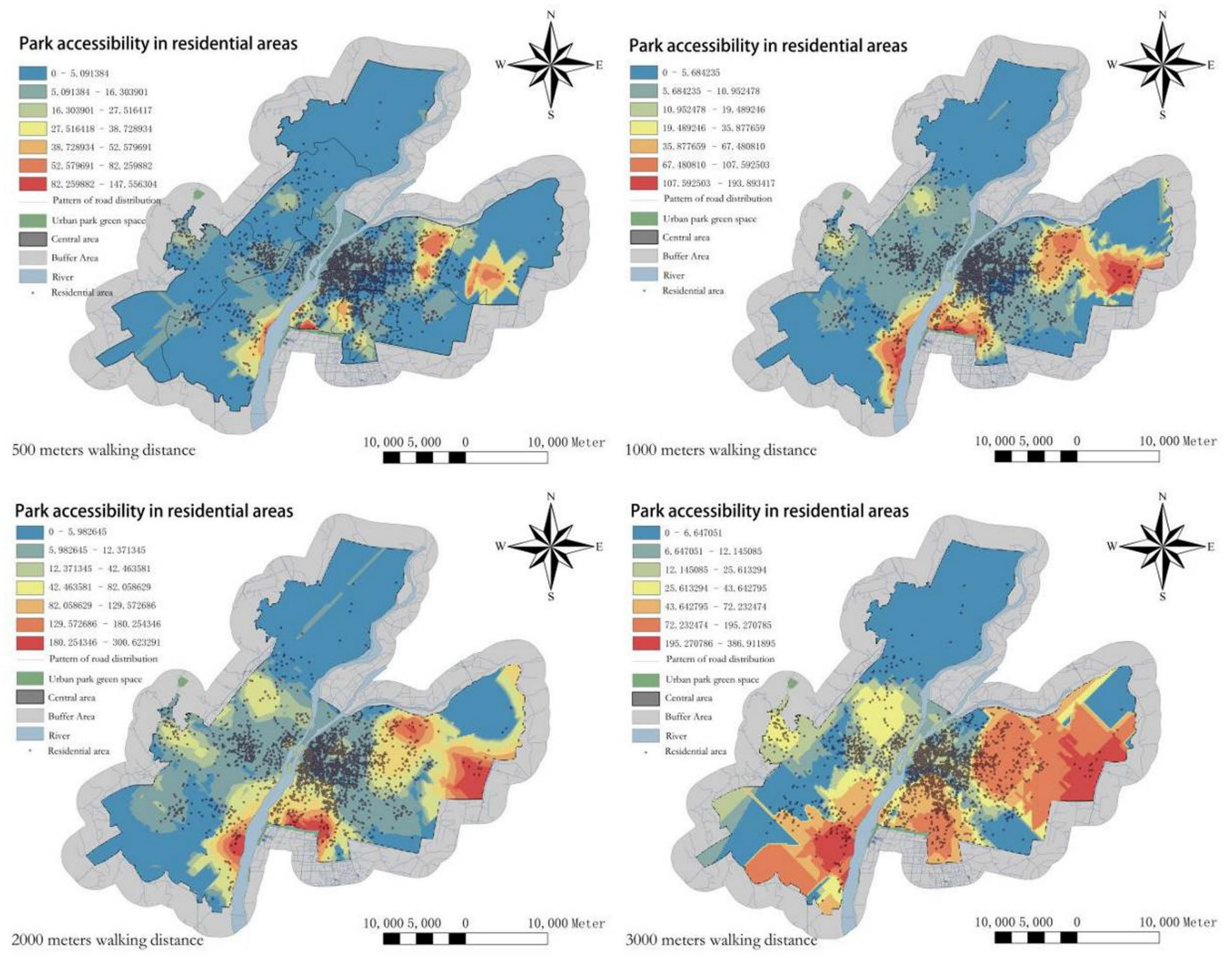
Spatial distribution of accessibility levels in different residential areas under different walking distances.

In this study, an improved GS2SFCA method is employed to calculate the accessibility of UPGS in Nanchang City. We collected accessibility indicators of residents at different walking distances (Table 2) and examined the distribution changes in park accessibility (Figure 3). The standard deviation in the table represents the degree of dispersion of UPGS accessibility, to some extent, indicating spatial equity (39).

**Table 2:** Accessibility description statistics of various residential areas under different walking distances.

Walking distance	UPGS accessibility			Number of settlements with 0 accessibility	Standard deviation
	Maximum	Minimum	Mean		
500	147.55	0	2.626891	2,138	14.881813
1,000	193.89	0	9.249018	1,013	28.890743
2000	300.62	0	23.440792	157	44.263746
3,000	389.91	0	35.715331	24	49.61402

Results show: (1) at a walking distance of 500 meters, over two-thirds of the residents have zero accessibility to UPGS, indicating the lowest overall accessibility. Areas with higher accessibility are concentrated in the southern and eastern edge regions. (2) When the walking distance increases to 1,000 meters, the number of residents with zero UPGS accessibility decreases significantly. However, the changes in UPGS accessibility in the old city area and the central along the Ganjiang River in Honggutan are still not pronounced. (3) With a walking distance of 2,000 meters, only one-twentieth of the residents have zero UPGS accessibility, and areas in the old city and Honggutan started to exhibit higher accessibility for residents. This suggests that the planned initial UPGS service range in Nanchang City is close to 2,000 meters, and the old city and Honggutan are two areas lacking internal UPGS supply. (4) When the walking distance extends to 3,000 meters, the number of residents with zero accessibility and the standard deviation decreases the least, while the average UPGS accessibility rises significantly. This indicates that changes in walking distance contribute the least to spatial equity at this stage. (5) In summary, UPGS accessibility for different walking distances exhibits a similar spatial pattern. The northern and southwestern parts of the city are cold spots, representing the main areas of explicit blind spots, while the eastern and southern regions are hot spots, where UPGS supply far exceeds residents’ demand. Increasing residents’ walking distance results in the spread of UPGS accessibility distribution from the periphery to the central area, indicating that increasing walking distance enhances UPGS accessibility. Accessibility is positively correlated with walking distance and the number of UPGS. The old city area and Honggutan, with the highest population density, require most residents to walk more than 2,000 meters to access UPGS. This indicates a severe supply–demand imbalance in these two regions and serves as the main areas of implicit blind spots. A walking distance of 2,000 meters provides the most significant improvement in spatial equity and can inform the determination of the radiation radius for additional UPGS at a later stage.

### 4.2 Overall accessibility disparity analysis

**Figure 4:**
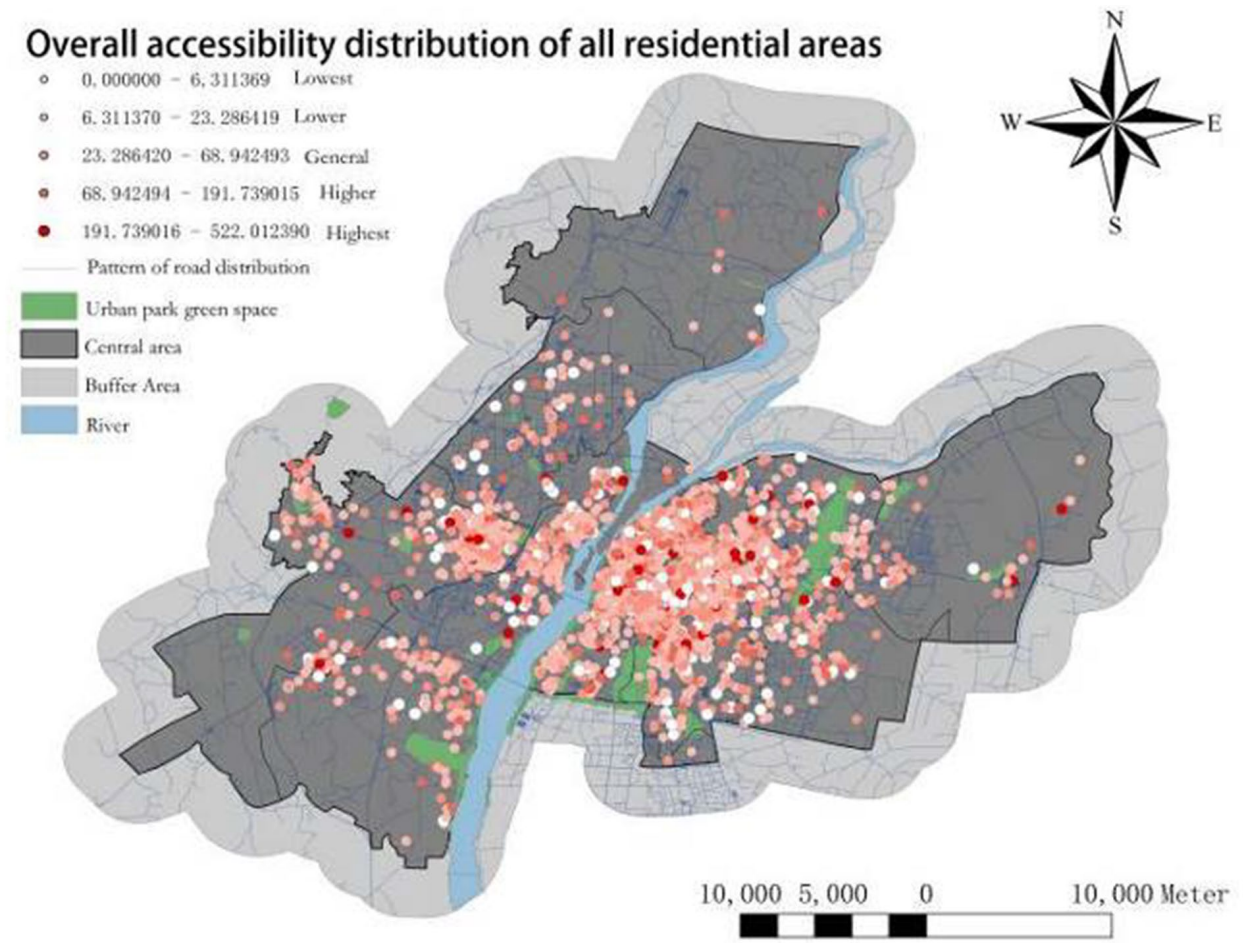
Overall accessibility distribution of all residential areas.

**Figure 5:**
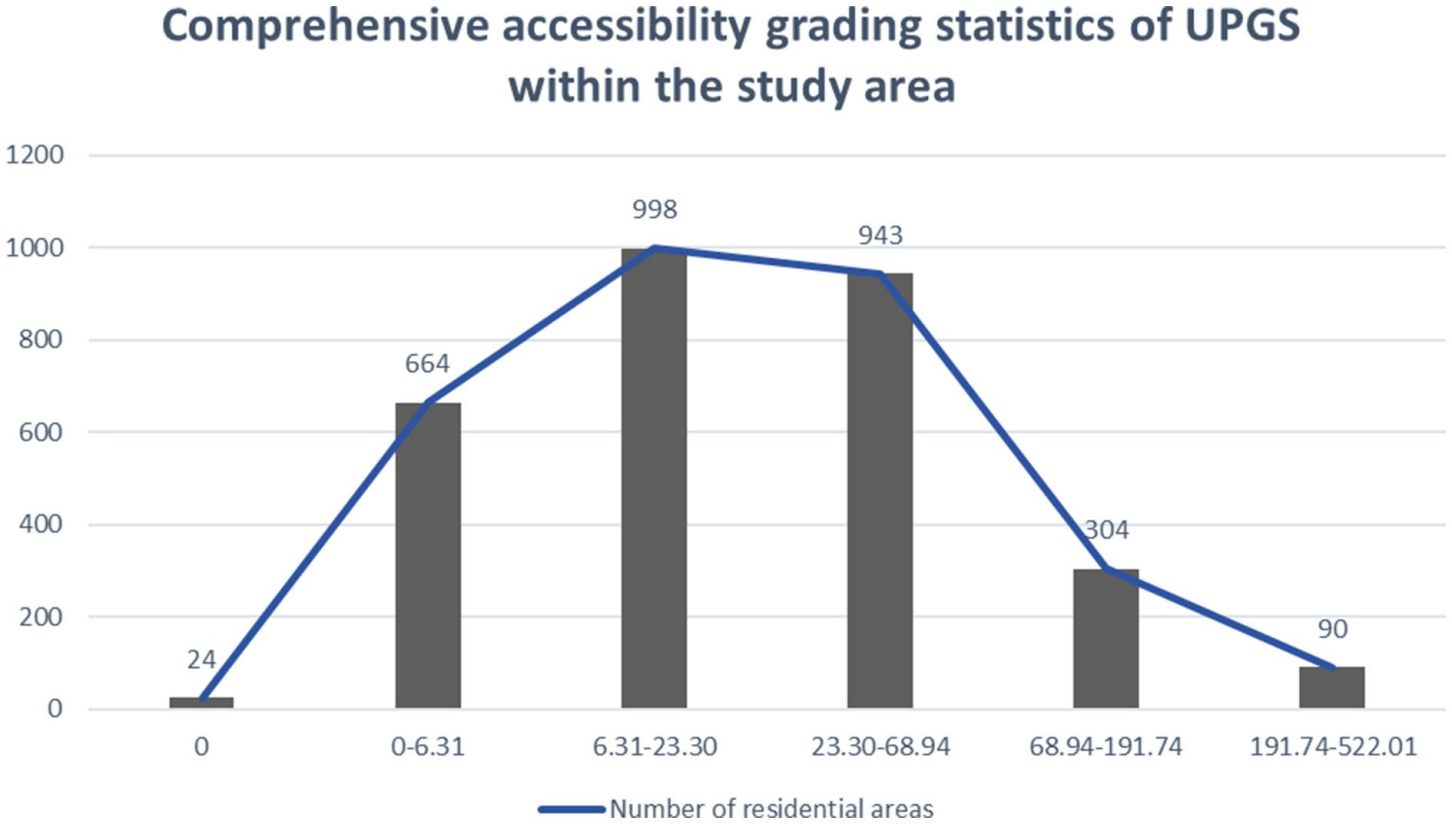
Comprehensive accessibility grading statistics of UPGS within the study area.

To visually represent the spatial distribution of overall accessibility in settlements, all UPGS accessibility data was overlaid using ArcMap 10.6 and divided into intervals using the geometric interval method to generate the UPGS comprehensive accessibility distribution layer (Figure 4) and hierarchical statistical chart (Figure 5), with settlements as units. In terms of geographic distribution, it can be found that the lowest accessibility settlements are mainly distributed in the fringe areas of settlement clusters, such as the northern part of Qingshanhu District and the southern part of Honggutan District. At the same time, the lower and medium accessibility settlements are mainly clustered in the old urban area and the opposite bank of Honggutan District. The possible reason for this is that residents on the edge of urban expansion have difficulties in having their needs for urban public infrastructure met by the government. The old city and Honggutan District, as the former and current development centers of Nanchang, are too densely populated, and demand exceeds supply, the population density directly affects the degree of accessibility. In addition, the settlements with higher accessibility on the map are mainly located around large UPGS. It may be that the population size of these settlements is smaller, and the large UPGS have more extensive areas, ESV, and FCs, resulting in an overall higher level of accessibility for these settlements as a whole. Overall, the total accessibility of the UPGS in the central region of Nanchang City shows a spatial pattern of decreasing towards the main area in the east, south, and west, consistent with the distribution of the large UPGS.

As depicted in Figure 5, only 24 settlements exhibit zero accessibility, indicating that the implementation of UPGS in Nanchang City has essentially extended to all accommodations, achieving geographical parity. However, it is noteworthy that a significant majority (over 80%) of settlements fall into the categories of low, lower, and general accessibility levels. This suggests a deficiency in adequate UPGS accessibility throughout Nanchang, resulting in spatial inequality where limited residents enjoy most UPGS resources. Conversely, settlements with high and higher accessibility levels comprise merely 304 and 90, respectively, but the quality of accessibility provision is much higher than the other types. In conclusion, while achieving geographic parity through existing UPGS construction has been accomplished mainly in Nanchang City, future optimization should focus on enhancing spatial equity and social fairness.

### 4.3 Analysis of social equity in UPGS

**Figure 6:**
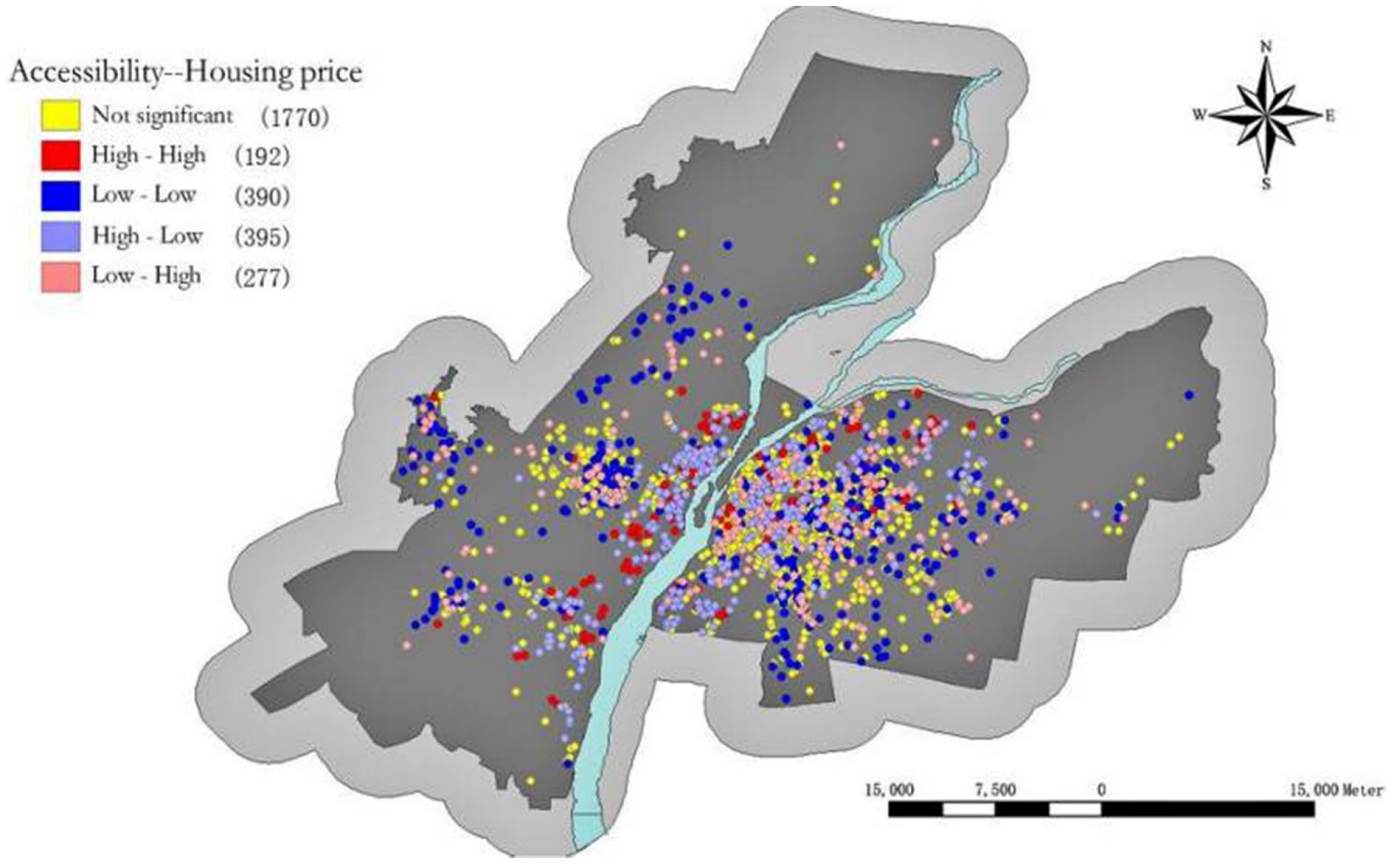
Spatial autocorrelation between accessibility and residents’ socioeconomic status.

The GeoDa software was utilized to investigate the spatial autocorrelation between the socioeconomic status of residents in the study area and UPGS accessibility. The binary global Moran index for their spatial coupling was 0.290, which passed the significance test at 0.01, indicating a positive overall spatial correlation (36). In other words, higher economic levels in an area corresponded to greater UPGS accessibility. Figure 6 illustrates a spatial mismatch between socioeconomic status and UPGS accessibility in Nanchang. Specifically, there were 192 high-high communities primarily concentrated in the southwest region near the river greenway, suggesting a shift in Nanchang’s economic development from its old urban area towards the southwest. Additionally, there were 390 low-low communities mainly located within the senior city center and on the outskirts of settlement clusters, aligning with areas exhibiting lower accessibility; this represents a significant imbalance in social equity. Furthermore, there were 395 high-low neighborhoods predominantly reliant on high-high communities due to their access to UPGS resources enjoyed by economically prosperous areas; finally, there were 277 low-high neighborhoods, which are more centrally distributed and spatially similar to the distribution of low-low communities.

## 5 UPGS accessibility optimization strategies

### 5.1 Analysis of adding UPGS quantity and site selection

**Figure 7:**
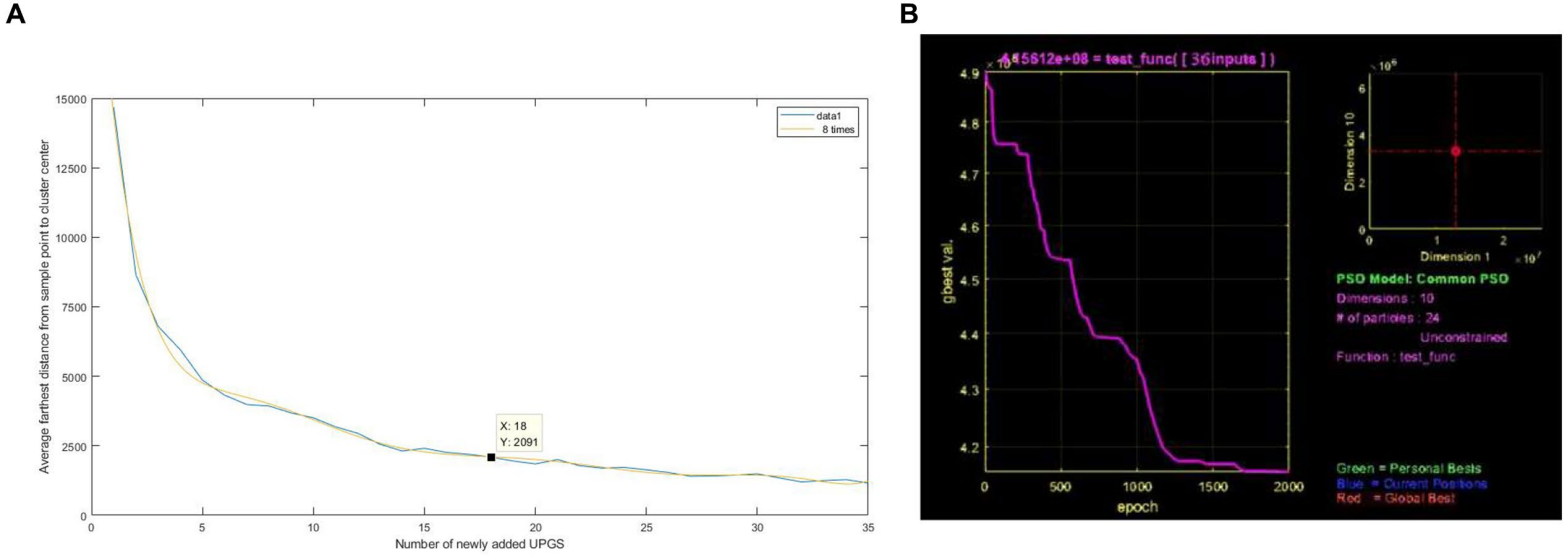
The UPGS quantity optimization curve **(A)** and the Iterative interface of PSO **(B)**.

To comprehensively balance the spatial equity and social equity of UPGS in Nanchang, 731 residential points that require improvement were identified by combining low accessibility residential points (0–6.311369) with low-low type communities that exhibit a socioeconomic mismatch. Subsequently, the K-means clustering algorithm in Matlab (67) was employed to determine the optimal number of UPGS through the maximum Euclidean distance in a two-dimensional space composed of accessibility and socio-economic adaptability levels of paired samples. After multiple iterations, the K-means clustering curve for optimizing UPGS in Nanchang was obtained (Figure 7A). The curve’s horizontal axis represents the number of newly added UPGS, the vertical axis represents the average farthest distance from sample points to cluster centers, and the slope of the curve indicates the impact of increasing the number of cluster centers on clustering effectiveness. Due to the construction of a “20 min walking time circle” and the standard deviation of the accessibility under different walking distances, it is finally confirmed that 18 new UPGS with a radial range of 2,000 m will be added based on the original one.

**Figure 8:**
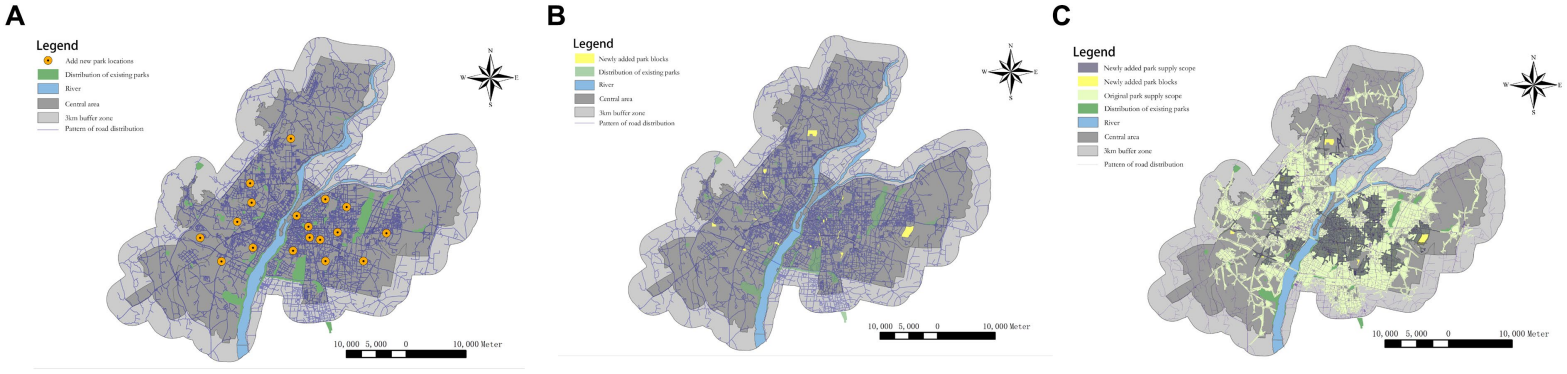
They added the UPGS point bitmap **(A)**, the area distribution of UPGS **(B)**, and added radiation range for UPGS **(C)**.

The K-means algorithm addresses the issue of determining the number of new UPGS but lacks specific location information to guide practical planning. To overcome this limitation, we employed the versatile and robust PSO, known for its effectiveness in highly nonlinear and discontinuous situations (68). Therefore, the PSO in Matlab was applied to take the spatial locations of the 18 UPGSs as the final output. The sum of the products of the population of a settlement with weak accessibility and the distance from that settlement to the nearest particle among the 18 particles was used as the objective function of optimization. The spatial information of the 18 new UPGS locations was finally obtained after nearly 2,000 iterations of the model (Figures 7B, 8A). However, the initially designated UPGS locations were all citywide parks with a radial range of 2,000 meters, which did not suit all areas of Nanchang. By integrating green space information from Nanchang’s latest planning blueprint, 18 citywide UPGS locations were selected as reference centers for positioning, ultimately yielding 12 community-type UPGS, 4 regional UPGS, and 13 citywide UPGS, totaling 29 UPGS (Figure 8B). Among these, community-type UPGS primarily target areas like the old city center characterized by complex land use and land scarcity, adopting an “acupuncture” approach to disperse resources and alleviate issues of high population density and associated social inequity driven by housing prices. Regional UPGS primarily comprise small greenways, enhancing landscape continuity by incorporating the city’s internal river network, and their distribution is relatively scattered. The citywide UPGS are situated in areas boasting natural beauty, convenient transportation, and a high concentration of nearby residents. They are primarily located at the periphery of residential clusters, eliminating implicit and explicit blind spots in accessibility and thus playing a crucial role in improving overall accessibility for residents. In summary, as evident from Figure 8C, the radiation ranges of the newly added UPGS substantially overlap with the existing UPGS, highlighting that this optimization primarily focuses on addressing implicit blind spots in accessibility, with the optimization concentrating on the old urban area and the northern part of the Qingshanhu District.

### 5.2 Optimization results analysis

**Figure 9:**
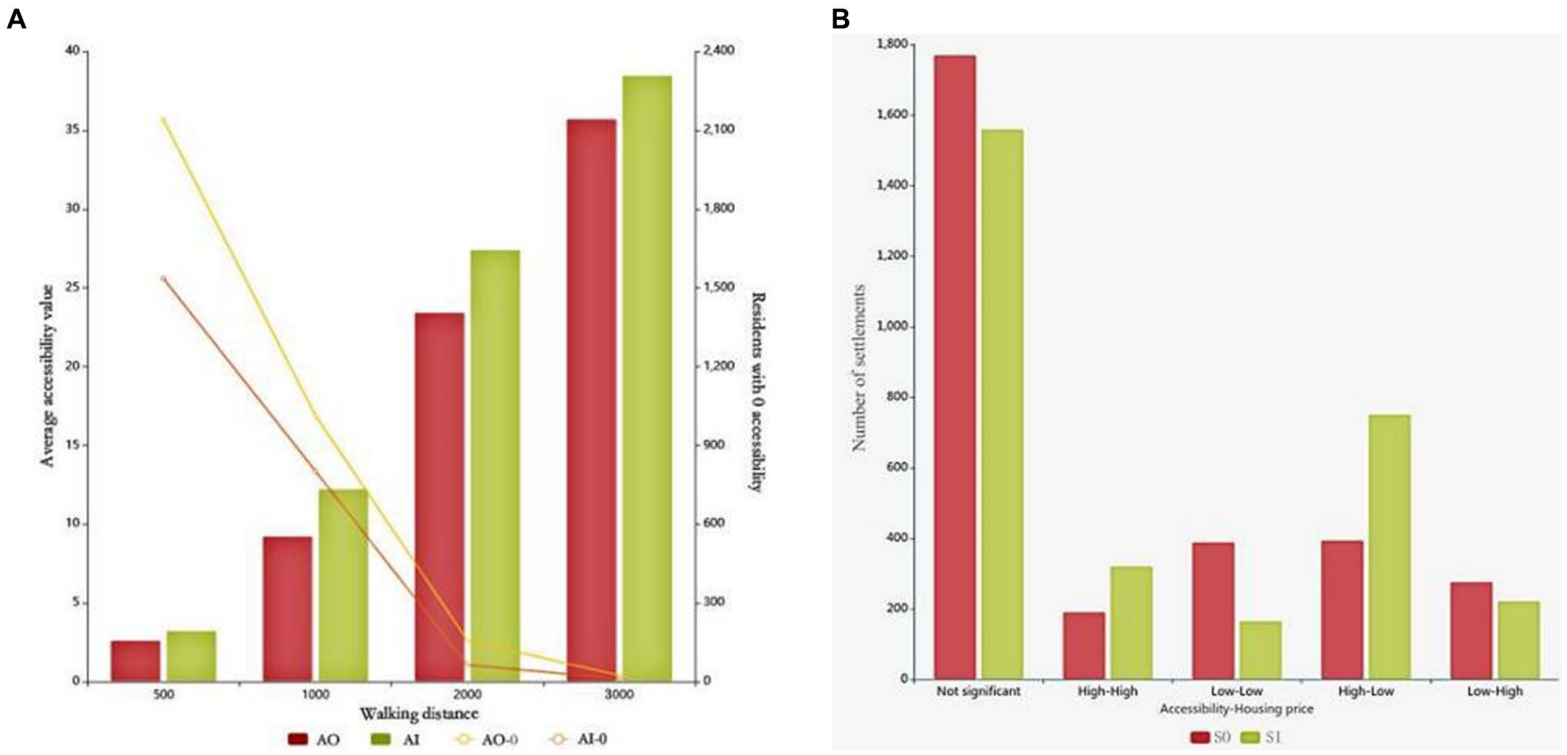
The comparison before and after UPGS optimization **(A)** and comparison of socioeconomic equity before and after UPGS optimization **(B)**.

Figure 9A shows the improvement of spatial equity in Nanchang by the new UPGS, which is the optimization of accessibility to both explicit and implicit blind zones, and the overall average accessibility rises by 2.76. However, the magnitude of the improvement varies across walking distances, with the new UPGS increasing the accessibility of residents at walking distances of 1,000 m and 2,000 m the most, followed by those at walking distances of 500 m and 3,000 m. This is because the new UPGS types are mainly citywide parks with a more extensive service radius and carrying capacity than community-based UPGS. In addition, for neighborhoods with 0 accessibility, the most excellent elimination occurs within a 500 m walking distance and then decreases as the distance increases. However, there are still 11 settlements with 0 accessibility not eliminated even after increasing to 3,000 m, which may be due to the poor connectivity of the road network in these settlements, which makes it difficult to solve the problem of accessibility provision by increasing the UPGS. For these suburban settlements with poor spatial connectivity, the focus should be on improving the service capacity of the transport road network, which will, in turn, improve the traveling efficiency and accessibility of the residents.

Figure 9B shows how the addition of UPGS has improved social equity in Nanchang City, with the most significant changes being in spatially mismatched low-low and high-low type neighborhoods. Specifically, the number of low-low-type communities decreased from 390 to 167, while the number of high-low-type communities increased from 395 to 752. These findings indicate a gradual reduction in the disparity of UPGS resources resident groups enjoy across different economic levels. Moreover, it highlights the significant impact of optimizing UPGS on enhancing social equity in Nanchang.

## 6 Discussion

This study explores the optimization of UPGS in Nanchang under the common goal of spatial and social equity and has three primary research outcomes in the process. The first is optimizing the accessibility calculation model using more objective and easier-to-calculate ESVs and FCs. The second is that the selection of optimization objectives considers both spatial and social fairness. The third is to combine the local green space planning of Nanchang City with the K-means algorithm and particle swarm algorithm to target the minimum walking distance and maximum fairness, and to propose a more suitable local optimization scheme for UPGS.

In the allocation of public resources between multiple cities, existing improvements to UPGS accessibility measurement models primarily focus on reducing resistance to accessibility by incorporating physical and environmental factors beyond city public space stations, while neglecting the influence of UPGS’s quality attributes (52, 69). However, a few studies on accessibility models based on quality attribute optimization often include subjective qualities, leading to significant cognitive biases in determining the importance of multiple factors (46). In contrast, we adopt the G2FCAS method with supply–demand improvement to evaluate the accessibility and fairness of UPGS. This approach introduces more objective factors on the supply side of UPGS, such as ESV and FCs, which are more focused on expressing “bottleneck” limiting factors related to visitor capacity. At the same time, quantifying these quality attributes differs from previous methods relying on expert evaluations or offline questionnaires (36, 39). Instead, it utilizes landscape pattern indices and hard surface area data as substitutes that are more transparent and easily accessible within UPGS. Therefore, this improved accessibility model ensures its potential replicability and convenience when applied in other cities. On the demand side of UPGS, the area and entrance information of the UPGS is obtained by web-crawled AOI combined with offline research to ensure the reliability of the data compared with manual depiction and purely offline survey (54, 58, 70); the population of the community is accepted as the product of the population of the administrative unit accurate to the neighborhood, and the number of households, instead of the population estimation of the homogenized distribution with the minimum unit of the district (71); and the socioeconomic level of the residents is obtained by using web-crawled community house prices as the estimation index, which ensures the heterogeneity and accuracy of each community point compared to some social equity studies that ignore group distribution patterns (45). In conclusion, the UPGS is statistically improved in both supply-side and demand-side measurements and data. However, in selecting optimization objectives, previous studies primarily focused on accessibility under spatial equity or accessibility under social equity, lacking a comprehensive consideration of both (23, 72). This study used 3,024 residential points as the foundational unit of data, overlaying both objectives for optimization, aiming to minimize computational costs while considering spatial fairness and social equity. Furthermore, this overlay optimization approach is not only applicable to the socio-economic perspectives investigated in this experiment but also extendable to other demographic groups with large population bases, such as educational attainment and gender. Subsequently, a more scientifically grounded approach was employed, combining the K-means algorithm and PSO with actual land use considerations, aiming for enhanced equity and minimized walking distance. Multiple iterations were conducted to determine the location and area of new UPGS with greater practical significance, and the validity of the optimization method was verified through measurement statistics. This study demonstrates both the feasibility and limitations of our accessibility measurement model within a research framework for evaluating UPGS accessibility and equity in Nanchang City; moreover, it highlights that this framework and methodology can be flexibly applied to other cities utilizing reasonable data.

It is worth noting that there is still considerable flexibility in determining the final area and location of the new UPGS. In addition to ensuring that the k-means algorithm results closely approximate the minimum distance in practical scenarios, it is essential to consider the following recommendations: (1) In densely populated and congested urban settings, new UPGS additions should primarily take the form of community parks, with a more significant proportion of hard surface area to enhance UPGS accessibility and tourist capacity. Additionally, addressing the pressure on UPGS supply can be achieved through developing rooftop gardens or sharing certain types of open spaces (such as those in communities and educational institutions) (73). (2) When the k-means algorithm results are close to existing UPGS locations, increasing the area of existing UPGS or modifying the internal hard surface areas can enhance park service capacity. Previous research has shown that transforming existing parks can enhance the fairness of park resource utilization (74). (3) For suburban residential areas with zero accessibility and poor urban connectivity, improving the service capacity of road networks is more effective than adding new UPGS. (4) Since the ESVs and FCs of UPGS also affect residents’ willingness to choose and limit the number of residents (3, 36), the service capacity of UPGS can all be enhanced by increasing the number, type, and patch quality of facilities within UPGS. Apart from the research outcomes, this paper has certain limitations. First, no established metric exists in model optimization to quantify the substitution effect of landscape pattern indices and hard surface area in place of ESV and FCs. Furthermore, the determination of the UPGS service radius was made hastily. Currently, mobile phone signal data can provide the movement trajectories of UPGS users, allowing for the identification of more precise UPGS service radii (20, 75). Third, other modes of transportation, such as cycling, public transit, automobiles, and subway travel (47), were not considered. Finally, there is still significant flexibility in determining the location of the new UPGS. Future research could potentially involve the development of an app that integrates local land use planning and input regarding existing UPGS supply and demand to calculate new UPGS location information directly.

## 7 Conclusion

In this study, taking Nanchang City as an example, we have developed a systematic procedure and framework for constructing the accessibility analysis and fairness evaluation and optimization of UPGS based on multi-source big data. By combining the improved G2SFCA model with K-means and PSO algorithms, we have obtained more accurate and, objective, and reasonable results. Through such objective factors as landscape pattern index and UPGS hard site area, the quality attributes ESV and FCs of the UPGS itself are estimated and incorporated into the existing GS2SFCA accessibility measurement model to comprehensively analyze the spatial distribution characteristics of the accessibility level of the UPGS in the study area. The results show that the distribution of UPGS accessibility in Nanchang is uneven, generally showing a spatial pattern of decreasing from east, south, and west towards the center. The spatial autocorrelation model between socioeconomic status and accessibility is used to uncover the degree of spatial coupling between the two. A significant positive correlation was found between socio-economic level and UPGS accessibility. On this basis, the community is used as a medium to couple the pending optimization objectives of spatial and social equity, and 29 new UPGS with specific locations and areas are obtained by combining the K-means algorithm with the particle swarm algorithm based on the land use data of Nanchang City. The optimization process is highly reliable and easy to operate and applies to the optimization of UPGS accessibility in other cities, which is beneficial for urban planners to develop effective improvement strategies for poorly served communities to achieve equity in the UPGS enjoyed by all residents within the city.,

## Data availability statement

The original contributions presented in the study are included in the article/supplementary material, further inquiries can be directed to the corresponding author.

## Author contributions

YZ: Conceptualization, Data curation, Formal analysis, Investigation, Methodology, Project administration, Software, Visualization, Writing – original draft, Writing – review & editing. PG: Funding acquisition, Resources, Supervision, Writing – review & editing.

## Footnotes

^1^ http://www.openstreetmap.org/

^2^ https://wuhan.anjuke.com/
